# Prevalence of Hypomineralized Second Primary Molar in Shaanxi, China

**DOI:** 10.3389/fpubh.2025.1703833

**Published:** 2025-11-12

**Authors:** Lamya Khalafalla Altieab Abdalla, Shijie Zhu, Pingneng Zhang, Peidi Huang, Kaixin Guo, Wenjiao Zhang, Bin Zhang, Xiao Hu, Ruizhe Huang

**Affiliations:** Key Laboratory of Shaanxi Province for Craniofacial Precision Medicine Research, College of Stomatology, Xi’an Jiaotong University, Xi’an, China

**Keywords:** Hypomineralized Second Primary Molar, prevalence, pregnancy vomiting, maternal education, pediatric dentistry

## Abstract

**Objectives:**

This cross-sectional study aimed to determine the prevalence of Hypomineralized Second Primary Molar (HSPM) in 3–5-year-old children in Shaanxi Province, China, and identify its associated factors, with a specific focus on exploring the potential role of pregnancy vomiting that have been less studied previously.

**Methods:**

Subjects were randomly sampled from three cities in Shaanxi Province, namely Xi’an, Yan’an, and Weinan. HSPM was evaluated through oral examinations, while other health-related data were gathered using questionnaires. Statistical analyses were performed using chi-square tests and binary logistic regression to identify independent risk factors and adjust for confounders.

**Results:**

A total of 1,022 questionnaires were distributed, with 1,015 valid ones collected from children in three cities. The sample included 48.97% males and 51.03% females, with regional distributions of 45.71% (Xi’an), 27.78% (Weinan), and 26.51% (Yan’an) of the sample. The overall prevalence of HSPM was 7.09%, showing differences by region and maternal education. Teeth with HSPM had a higher caries rate (55.6%) than those without (45.3%), though the difference was not significant. Univariate analysis linked HSPM to gestational age (*p* = 0.022), asthma in the first year of life (*p* = 0.03), measles (*p* = 0.003) and maternal nausea (*p* = 0.018), in the adjusted multivariable model, maternal education (bachelor’s degree, OR = 0.297, 95%CI: 0.122, 0.72) and maternal vomiting (OR = 1.694, 95%CI: 1.027, 2.795) associated with HSPM.

**Conclusion:**

The prevalence of HSPM in Shaanxi is within the global range, while maternal lower education level and pregnancy vomiting are key risk factors for HSPM. These findings provide novel insights for targeted prenatal interventions to prevent HSPM in preschool children.

## Introduction

Enamel, the hardest mineralised tissue in the human body, cannot regenerate after formation as ameloblasts (cells responsible for enamel secretion) disappear post-development (1). Disrupted ameloblast activity during tooth development leads to enamel abnormalities: enamel hypoplasia (quantitative defect from impaired matrix deposition) and enamel hypomineralisation (qualitative defect from disturbed mineralisation). Hypomineralised enamel has structural defects, making it more prone to chipping and enamel loss after eruption (2), which is being relevant to the study of Hypomineralized Second Primary Molar (HSPM) (3, 4).

HSPM is a systemic, multifactorial enamel mineralisation defect primarily affecting second deciduous molars (5), with a global prevalence of 4.9–9.0% (6). It weakens enamel structure and increases caries susceptibility, differing from permanent teeth-specific Molar Incisor Hypomineralisation (MIH) (7). MIH refers to insufficient enamel mineralisation in at least one first permanent molar, often accompanied by less severe defects in the incisors. Globally, the prevalence of MIH varies significantly across regions, it ranges from 2.3% in some parts of Africa (8) to 10% in Beijing (9), China. Importantly, the enamel defects in HSPM and MIH are highly similar, typically asymmetric, and manifest as distinct white to pale yellow or brownish spots. In both conditions, the enamel in affected areas has lower hardness, poorer elasticity, and reduced aesthetic appeal (10, 11). While global HSPM prevalence data exist, regional studies in China—especially in Shaanxi Province, a region with distinct socioeconomic and demographic characteristics—remain scarce. This gap hinders the development of region-specific oral health strategies, highlighting the need for local epidemiological data.

Primary molars are the main site for HSPM observation, which is a critical predictor of MIH in permanent teeth (12–14). For example, in Israel, the prevalence of HSPM in children was reported to be 6.0%, while the prevalence of MIH was significantly higher at 10.3% (12). Specifically, studies have shown that hypomineralization of the deciduous molars is associated with a higher probability of developing MIH at 7–8 years of age (15, 16), furthermore, MIH is, in turn, associated with an increased risk of dental caries, both in the deciduous and permanent dentition (17). No comparable data exist for Shaanxi, making local investigation imperative. Existing studies link HSPM to prenatal (e.g., maternal alcohol use), perinatal (e.g., preterm birth), and postnatal (e.g., childhood fever) factors (6) highlighting the need to explore these domains comprehensively. Notably, maternal pregnancy-related symptoms like nausea and vomiting—potentially linked to nutrient absorption and fetal enamel development—have rarely been investigated as HSPM risk factors, representing a key research gap (18). Evidence suggests that HSPM and MIH risk factors can be classified as prenatal, perinatal, or postnatal. Prenatal factors include pregnancy issues, maternal alcohol use or smoking, and antibiotic use. Perinatal factors include preterm birth, mode of delivery, neonatal hypoxia or respiratory issues, birth weight, and insufficient vitamin D intake. Evidence indicates that high-dose vitamin D supplementation during pregnancy is a protective factor against enamel hypomineralization in both deciduous and permanent dentition, highlighting the importance of adequate vitamin D levels during this period (19). Postnatal factors include breastfeeding status; childhood asthma; high fever; various infections/diseases (such as chickenpox, diarrhoea, pneumonia); and antibiotic use (20). MIH is diagnosed from 7 years of age onwards, when the first permanent molars have erupted. According to pediatric dentistry associations, the optimal age for MIH diagnosis is between 7 and 9 years; it is after diagnosis that the most severe consequences of MIH can be observed in children. This highlights the need for early intervention targeting HSPM risk factors to mitigate potential MIH-related issues (21). The main aim of this cross-sectional study is to: (1) determine HSPM prevalence in 3–5-year-old children in Shaanxi Province; (2) identify associated prenatal, perinatal, and postnatal risk factors, with a focus on maternal pregnancy vomiting (a understudied factor). This study will fill these gaps and provide targeted scientific evidence for HSPM prevention and early intervention in local preschool children.

## Materials and methods

### The sample

This cross-sectional survey was conducted from November 2024 to January 2025 in Shaanxi Province, using stratified cluster random sampling. Kindergartens were randomly selected from three cities (Xi’an, Yan’an, Weinan) based on urban/rural distribution, with all children from the selected kindergartens included in the sample. All children meeting the following criteria were included in the study: (1) age range: 3–5 years old; (2) Chinese students born and residing in Shaanxi Province; (3) at least one deciduous molar has fully erupted or partially erupted; (4) no history of long-term medication use; (5) no history of systemic diseases or syndromes; (6) normal saliva flow. It was approved by the Ethics Committee of the School of Stomatology, Xi’an Jiaotong University (Approval Number: KY-QT-20240015).

### Survey questionnaire

A structured questionnaire-adapted from validated instruments, pre-tested on 50 mother–child pairs, and yielding a Cronbach’s *α* coefficient of 0.82 for internal consistency—and a clinical examination form were employed for standardized data collection. The 14 structured questionnaire items targeted mothers of 3–5-year-old children, covering: demographic information (child’s age, maternal education), birth-related factors (birth weight, gestational age), child’s first-year health (infections, hospitalisation), and maternal pregnancy health (medication use, vomiting). To facilitate the data collection process, the kindergarten played a pivotal role in distributing the questionnaires and informed consent forms to the parents. We emphasized the importance of informed consent, and all parents who agreed to participate in the survey were required to sign the consent form.

### Clinical diagnosis

**Figure 1:**
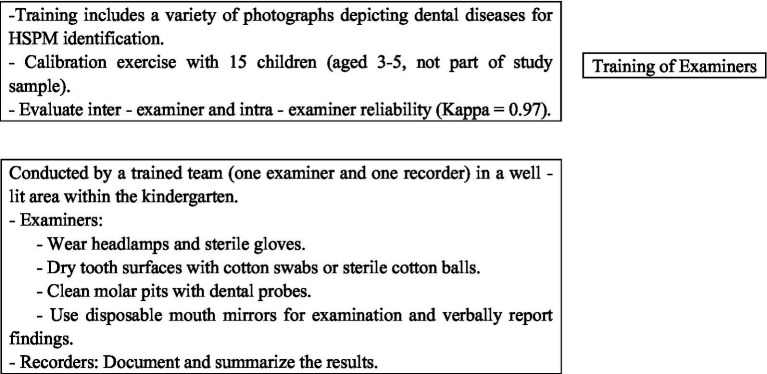
Details of clinical diagnosis process.

**Figure 2:**
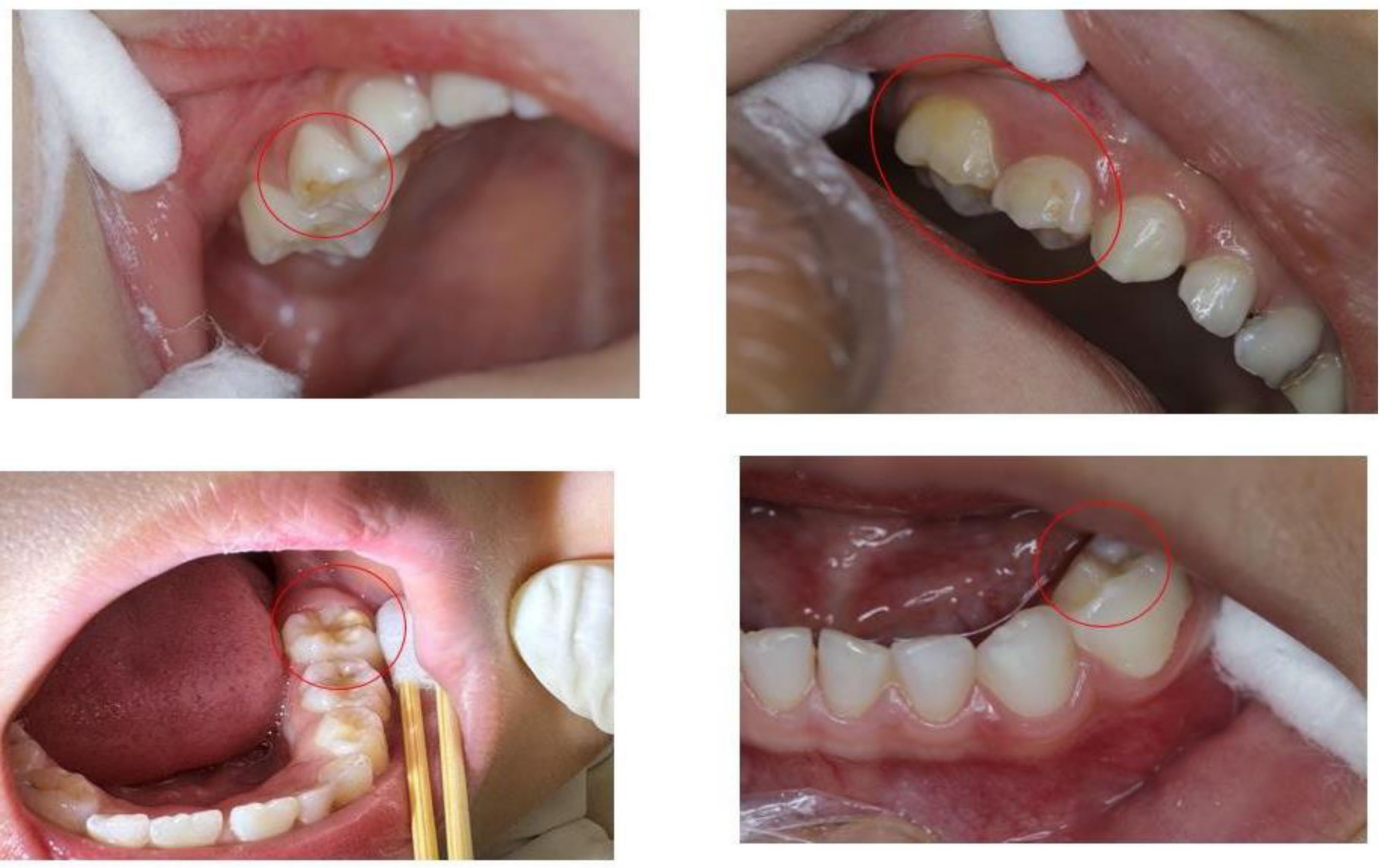
Intraoral photographs of typical HSPM symptoms.

Experienced dentists provided theoretical training to two examiners responsible for conducting all clinical evaluations. This training included a variety of photographs depicting dental diseases to aid in the identification of HSPM. Additionally, a calibration exercise was carried out with a group of 15 children between the ages of 3 and 5 years, who were not part of the study sample. After this exercise, both inter-examiner and intra-examiner reliability were evaluated and determined to be high (Kappa = 0.97). All clinical assessments were conducted by the same trained team (comprising one examiner and one recorder) in a designated, well-lit area within the kindergarten. The specific process details are shown in Figure 1. The HSPM assessment is based on the standards of the European Academy of Paediatric Dentistry (EAPD) and is conducted by Figure 2.

### Statistical analysis

Data analysis used SPSS 27.0. Descriptive statistics and chi-square tests compared study variables. Probability values less than 0.05 indicated statistical significance. Strength of associations was assessed with unadjusted and adjusted odds ratios (OR), 95% confidence intervals (CI), and *p*-values (Wald test). Univariate regression evaluated the influence of each independent variable with HSPM present. Variables were included in the final multiple model if statistically significant (*p* < 0.20) or clinically important. In the adjusted model, variables associated with HSPM (*p* < 0.05) or those improving model fit were retained. Odds ratios and 95% confidence intervals were calculated.

## Results

A total of 1,015 3–5-year-old preschool children were included, with 48.97% (497) males and 51.03% (518) females. Geographically, 45.71% (464) were from Xi’an, 27.78% (282) from Weinan, and 26.51% (269) from Yan’an. The overall HSPM prevalence was 7.09% (72/1015), with higher rates in males (8.70%) than females (5.60%) and lower rates in Xi’an (4.70%) than Weinan (9.90%) and Yan’an (8.20%). Maternal education was associated with HSPM, with prevalence decreasing from 14.6% (junior high or below) to 4.9% (bachelor’s degree) (see Table 1).

**Table 1:** Distribution of HSPM by age, location and mother’s educational attainment.

Variables	With HSPM	Without HSPM	Total	*χ* ^2^	*p* value
	*n* (%)	*n* (%)	*n* (%)		
Gender					
Male	43 (8.7%)	454 (91.3%)	497 (48.97%)	3.59	0.058
Female	29 (5.6%)	489 (94.4%)	518 (51.03%)		
Location					
Xi’an	22 (4.7%)	442 (95.3%)	464 (45.71%)	7.82	0.020
Weinan	28 (9.9%)	254 (90.1%)	282 (27.78%)		
Yan’an	22 (8.2%)	247 (91.8%)	269 (26.51%)		
Mother’s educational attainment					
Junior high school or below	13 (14.6%)	76 (85.4%)	89 (8.77%)	13.33	0.004
High school/college	27 (9.0%)	273 (91.0%)	300 (29.56%)		
Bachelor’s degree	26 (4.9%)	508 (95.1%)	534 (52.61%)		
Master’s degree and doctorate	6 (6.5%)	86 (93.5)	92 (9.06%)		
Caries					
Yes	40 (55.6%)	427 (45.3%)	467 (46.01%)	2.843	0.092
No	32 (44.4%)	516 (54.7)	548 (53.99%)		
Total			1,015 (100%)		

Table 2 shows the univariate regression results for the association between HSPM and prenatal, perinatal, and postnatal variables. HSPM was associated with gestational age (*p* = 0.022), asthma in the first year of life (*p* = 0.03), measles (*p* = 0.003), jaundice (*p* = 0.065), maternal nausea during pregnancy (*p* = 0.018), fever during pregnancy (*p* = 0.13), and alcohol consumption during pregnancy (*p* = 0.124). No significant associations were found with mode of delivery, birth weight, other diseases within the first year, antibiotic use, or maternal factors such as diarrhoea, colds, antibiotic use, hypertension, and diabetes during pregnancy.

**Table 2:** Frequency distribution and odds ratio of HSPM according to prenatal, perinatal, and postnatal factors.

Variables	With HSPM	Without HSPM	OR	95%CI	*p* value*
Type of delivery					
Vaginal delivery	43	516	1		
Caesarean section	29	427	0.815	(0.5, 1.328)	0.411
Gestational age					
Less than 37 weeks	9	53	1		
37–40 weeks	63	890	0.417	(0.197, 0.884)	0.022*
Birth weight					
Less than 2,500 g	70	883	1		
More than 2,500 g	2	60	2.378	(0.569, 9.935)	0.235
Twins					
No	71	924	1		
Yes	1	19	0.685	(0.09, 5.191)	0.714
Hospitalised within the first week of life due to illness					
No	65	856	1		
Yes	7	87	1.06	(0.471, 2.382)	0.889
Frequent high fever within the first year of life					
No	52	734	1		
Yes	20	209	1.351	(0.789, 2.314)	0.273
Tonsillitis within the first year of life					
No	67	847	1		
Yes	5	96	0.658	(0.259, 1.674)	0.38
Pneumonia within the first year of life					
No	65	862	1		
Yes	7	81	1.146	(0.509, 2.582)	0.742
Asthma within the first year of life					
No	70	939	1		
Yes	2	4	6.707	(1.207, 37.257)	0.03*
Otitis media within the first year of life					
No	70	932	1		
Yes	2	11	2.421	(0.526, 11.136)	0.256
Chickenpox within the first year of life					
No	71	940	1		
Yes	1	3	4.413	(0.453, 42.973)	0.201
Measles within the first year of life					
No	69	939	1		
Yes	3	4	10.207	(2.239, 46.516)	0.003*
Jaundice within the first year					
No	42	650	1		
Yes	30	293	1.585	(0.972, 2.582)	0.065
Eczema within the first year					
No	54	724	1		
Yes	18	219	1.102	(0.633, 1.918)	0.731
Rubella within the first year					
No	71	929	1		
Yes	1	14	0.935	(0.121, 7.21)	0.948
Urinary tract infection within the first year					
No	72	918	1		
Yes	0	25	0	0	0.998
Kidney disease within the first year					
No	72	942	1		
Yes	0	1	0	0	1
Mumps within the first year					
No	71	942	1		
Yes	1	1	13.268	(0.821, 214.35)	0.069
Scarlet fever within the first year					
No	72	942	1		
Yes	0	1	0	0	1
Vomiting and diarrhoea within the first year					
No	57	747	1		
Yes	15	196	1.003	(0.556, 1.81)	0.992
Antibiotic use within the first year					
No	67	857	1		
Yes	5	86	0.744	(0.292, 1.895)	0.535
Nausea during pregnancy					
No	43	687	1		
Yes	29	256	1.81	(1.106, 2.961)	0.018*
Diarrhoea during pregnancy					
No	904	67	1		
Yes	39	5	1.73	(0.66, 4.534)	0.265
Common cold during pregnancy					
No	55	739	1		
Yes	17	204	1.12	(0.636, 1.971)	0.695
Fever during pregnancy					
No	67	911	1		
Yes	5	32	2.125	(0.802, 5.63)	0.13
Alcohol consumption during pregnancy					
No	71	941	1		
Yes	1	2	6.627	(0.594, 73.97)	0.124
Hypertension during pregnancy					
No	71	924	1		
Yes	1	19	0.685	(0.09, 5.191)	0.714
Diabetes during pregnancy					
No	70	911	1		
Yes	2	32	0.813	(0.191, 3.464)	0.78
Antibiotic use during pregnancy					
No	66	891	1		
Yes	4	52	1.038	(0.364, 2.959)	0.944

OR, Odds Ratio; ^*^statistically significant *p*-value <0.05.

After excluding variables with weak associations (*p* > 0.2), five factors (birthplace, maternal education, gestational age, jaundice history, pregnancy vomiting) were included in the final model. Results showed maternal education (bachelor’s degree: OR = 0.297, 95% CI: 0.122–0.72) and pregnancy vomiting (OR = 1.694, 95% CI: 1.027–2.79) were independently associated with HSPM, while birthplace, gestational age, and jaundice history were not (Table 3). Variables with small positive case numbers (asthma, measles, pregnancy fever, alcohol consumption) were excluded from multivariable regression to avoid model divergence/large standard errors, though this may introduce selection bias (discussed in Limitations).

**Table 3:** Ratio of association between HSPM and adjusted independent variables and confidence intervals.

Variables	Adjusted OR	95%CI	*p* value*
Location			
Xi’an	1		
Weinan	1.267	(0.626, 2.563)	0.511
Yan’an	1.783	(0.952, 3.339)	0.071
Mother’s educational attainment			
Junior high school or below	1		0.058
High school/college	0.546	(0.257, 1.161)	0.116
Bachelor’s degree	0.297	(0.122, 0.72)	0.007*
Master’s degree and doctorate	0.362	(0.112, 1.17)	0.09
Gestational age			
Less than 37 weeks	1		
37–40 weeks	0.503	(0.234, 1.081)	0.078
Jaundice within 1 year of birth			
No	1		
Yes	1.561	(0.947, 2.574)	0.081
Pregnancy vomiting			
No	1		
Yes	1.694	(1.027, 2.795)	0.039*

OR, Odds Ratio; *statistically significant *p*-value <0.05.

## Discussion

This study assessed HSPM prevalence and associated factors in Shaanxi, China. We find an overall prevalence of 7.09%, which is not an extremely rare condition in this group but rather a relatively common oral health issue that requires attention (22). It aligns with the reported range of 4.9–9.0% in a recent meta-analysis (20). It is also comparable to the recently reported prevalence rate in northern India (7.51%) (23) and Israel (6.0%) (12). Any pathology with a prevalence greater than 5% is classified as significant and holds epidemiological importance (12).

Our study reveals a significant association between maternal educational attainment and HSPM (*p* = 0.004). Previous studies investigating the relationship between maternal educational attainment as a prenatal factor and HSPM have not found a significant association between the two, regardless of whether a simple dichotomous classification based on 8 years of education was used (24) or a more detailed classification based on primary school, secondary school, vocational school, university, and postgraduate education was employed (25). In this study, the classification strategy for maternal educational attainment was adjusted, dividing it into four levels: junior high school or below, senior high school and vocational school, bachelor’s degree, and master’s/doctoral degree. After adjusting for confounding factors, the analysis indicated that children of mothers with a bachelor’s degree had a significantly lower risk of developing HSPM compared to those of mothers with a junior high school education or below (OR = 0.297; 95% CI: 0.122–0.72). Mothers with higher education tend to have better health literacy (26), enabling them to access and understand prenatal care guidelines, such as balanced nutrition intake and avoidance of harmful behaviors, which are critical for fetal enamel development. The non-significant protective effect of a master’s/doctoral degree may be due to small sample size or confounding factors like work-related stress leading to compromised prenatal care, warranting further investigation.

To our knowledge, this is one of the first epidemiological studies to identify pregnancy vomiting as an independent risk factor for HSPM (OR = 1.694, 95% CI: 1.027–2.795), a novel finding that adds to HSPM aetiological understanding. Previous research has examined the associations between fever during pregnancy, maternal alcohol consumption, smoking, and antibiotic use with HSPM and MIH (20). However, the relationship between nausea and vomiting during pregnancy and HSPM has not been comprehensively investigated. The present findings indicate that pregnancy-related vomiting may contribute to the development of HSPM. A plausible mechanism is that severe/persistent vomiting (e.g., hyperemesis gravidarum) reduces maternal nutrient intake/absorption, leading to deficiencies in folate, vitamin D, or essential fatty acids—nutrients critical for fetal enamel development. Notably, pregnancy vomiting data were self-reported by mothers, which may introduce recall bias. Several studies have reported a significant association between gestational age and both MIH and HSPM (20, 27, 28). Primary tooth buds develop during the second month of embryonic growth and begin calcification between the fifth and sixth months. Preterm birth can disrupt the calcification of primary tooth buds, potentially resulting in HSPM. In the present study, no association was identified between preterm birth and HSPM, which aligns with the findings of Allazzam et al. (25). After adjusting for confounding variables, a history of jaundice within the first year of life was not significantly associated with HSPM, and the relationship between neonatal jaundice and HSPM remains inconclusive.

## Conclusion

This study found a 7.09% HSPM prevalence in 3–5-year-old children in Shaanxi Province, within the global range. Key findings include: (1) maternal bachelor’s degree is a protective factor against HSPM; (2) pregnancy vomiting is an independent risk factor for HSPM. These results highlight the importance of integrating maternal education and prenatal symptom management into HSPM prevention strategies. Future research should explore the mechanism of pregnancy vomiting on HSPM and validate these findings in larger cohorts. When investigating HSPM pathogenesis, comprehensive consideration of demographic (e.g., maternal education) and prenatal (e.g., pregnancy vomiting) factors is essential.

### Limitation

This study has several main limitations. The research concentrates on the prevalence of HSPM in preschool children within Shaanxi Province. Subsequent studies should consider the factors that impact the varying index of HSPM in this population. The inclusion of pregnancy vomiting as a variable has advanced understanding of the complex aetiology of HSPM. However, the present study did not stratify vomiting severity (e.g., frequency, duration), so the dose–response relationship between vomiting severity and HSPM remains unclear, warranting further investigation.

## Data Availability

The data analyzed in this study is subject to the following licenses/restrictions: the raw data supporting the conclusions of this article will be made available by the authors, without undue reservation. Requests to access these datasets should be directed to 2443531165@qq.com.
